# Vitamin D attenuates inflammation, fatty infiltration, and cartilage loss in the knee of hyperlipidemic microswine

**DOI:** 10.1186/s13075-016-1099-6

**Published:** 2016-09-13

**Authors:** Vikrant Rai, Nicholas E. Dietz, Matthew F. Dilisio, Mohamed M. Radwan, Devendra K. Agrawal

**Affiliations:** 1 Department of Clinical and Translational Science, Creighton University School of Medicine, CRISS II Room 510, 2500 California Plaza, Omaha, NE 68178 USA; 2 Department of Pathology, Creighton University School of Medicine, 601 North 30th Street, Omaha, NE 68131 USA; 3 Department of Orthopedic Surgery, Creighton University School of Medicine, Omaha, NE 68178 USA; 4 CHI Health Alegent Creighton Clinic, 601 North 30th Street, Suite 2300, Omaha, NE 68131 USA

**Keywords:** Osteoarthritis, Vitamin D deficiency, Cartilage loss, Inflammation, Fatty infiltration, Vitamin D supplementation, Inflammation attenuation

## Abstract

**Background:**

Osteoarthritis (OA) of the knee joint is a degenerative process resulting in cartilage loss. Recent evidence suggests that OA is not merely a disease of cartilage but a disease of the entire knee joint and that inflammation may play an important role. OA has been associated with vitamin D deficiency. Vitamin D as an immunomodulator and anti-inflammatory agent may attenuate inflammation in the knee. The aim of this study was to assess the anti-inflammatory effect of vitamin D on inflammation in the knee.

**Methods:**

This study was conducted with 13 microswine on a high cholesterol diet categorized into three groups of vitamin D-deficient, vitamin D-sufficient, and vitamin D supplementation. After 1 year, microswine were killed, and their knee joint tissues were harvested. Histological and immunofluorescence studies were carried out on the tissue specimens to evaluate the effect of vitamin D status.

**Results:**

Histological and immunofluorescence studies of the knee joint tissues showed (1) increased inflammation in the knee joint tissues, (2) fatty infiltration in quadriceps muscle, patellar tendon, and collateral ligaments, and (3) chondrocyte clustering in the vitamin D-deficient and vitamin D-sufficient groups compared with the vitamin D supplementation group. Architectural distortion of the quadriceps muscle, patellar tendon, and collateral ligaments was also seen in the areas of inflammatory foci and fatty infiltration in the vitamin D-deficient group.

**Conclusions:**

Decreased inflammation and fatty infiltration in the vitamin D supplementation group suggest the potential role of vitamin D in attenuating inflammation and fatty infiltration as well as in protecting the architecture of the tissue in the knee joint.

**Electronic supplementary material:**

The online version of this article (doi:10.1186/s13075-016-1099-6) contains supplementary material, which is available to authorized users.

## Background

Inflammation of the joint accompanied with pain and swelling is a major problem leading to joint damage. This inflammation is accompanied with loss of joint flexibility and range of motion, resulting in physical disability and limitation of movement. Among the hand, hip, and knee joints, knee joint arthritis is the most common form of arthritis [1]. Osteoarthritis (OA) of the knee joint is a degenerative joint disease that is the foremost cause of disability in the elderly population in the United States [2]. OA is characterized by an imbalance between degenerative and regenerative processes, resulting in cartilage loss [3]. Although the pathogenesis of cartilage degeneration is still unclear, there is evidence which suggests that cartilage damage is due to periarticular bone resorption and sclerosis during subchondral bone remodeling. This subchondral bone remodeling response may be slowed by low levels of vitamin D, resulting in bone thickening, osteophyte formation, and resultant cartilage damage [4, 5]. Therefore, low levels of vitamin D may affect structural changes of the knee joint [3]. OA affects all the knee joint tissues, including cartilage, muscle [2, 6], tendon, ligament, and subchondral bone, and these changes play an important role in the pathogenesis of OA [7]. Vitamin D influences the state of these articular structures by acting through vitamin D receptors (VDRs). *VDR* polymorphism has been associated with OA, and therefore vitamin D may play an important role in OA pathogenesis [8–10].

Vitamin D deficiency is common worldwide [11]. Vitamin D deficiency has been associated with many musculoskeletal diseases, such as muscle weakness, rickets, osteomalacia, osteopenia, and osteoporosis, as well as increased risk of fracture and muscle weakness [12]. The important role of vitamin D in bone mineralization, remodeling, and maintenance is well known, but the role of vitamin D in the pathogenesis of OA is yet to be defined [4]. Low levels of vitamin D are associated with progression and increased prevalence of OA [13–16]. Many studies support the beneficial role of vitamin D in OA [17, 18], but this is controversial [19, 20]. Low levels of vitamin D have also been associated with an increased incidence of inflammation [21, 22]. Recent evidence suggests a potential role of inflammation in OA pathogenesis [23, 24], and vitamin D as an immunomodulatory and anti-inflammatory agent may attenuate inflammation in the knee.

Macrophages are potent modulators of inflammation and, as sentinels of the innate immune system, are involved in the inflammatory response. OA is a wear-and-tear disease, and wear particles also stimulate a macrophage response [25]. Macrophages and macrophage-produced cytokines play a potential role in the pathogenesis of OA [26]. Thus, inflammatory mediators or markers expressed on macrophages may play a role in the pathogenesis of OA. Triggering receptor expressed on myeloid cells (TREM)-1 is a recently discovered amplifier of inflammation expressed on monocytes and/or macrophages and neutrophils, and TREM-2, an anti-inflammatory marker secreted from macrophages and dendritic and microglial cells, plays a key role in many inflammatory diseases [27–29]. TREM-1 plays a potential role in the pathogenesis of rheumatoid arthritis [30]. However, the role of TREM-1 and TREM-2 in OA is largely unknown. Further, early innate response due to trauma to the joint results in secretion of adiponectin and leptin by adipose tissue [7, 31–33]. The effect of vitamin D status on release of these adipokines in inflamed knee joints is largely unknown. Because vitamin D is an immunomodulatory and anti-inflammatory agent, vitamin D supplementation may affect the expression of TREM-1, TREM-2, adiponectin, and leptin, but this association is currently not well defined.

Vitamin D deficiency and decreased expression of *VDR* are associated with increased inflammation of epicardial fat, and vitamin D supplementation reduces this inflammation [34]. Further, hyperlipidemia and high fructose are also instigators of inflammation [34, 35]. While studying the effect of vitamin D status on the development of atherosclerotic lesions in the coronary arteries of swine fed a high-cholesterol and high-fat diet, we observed increased inflammation in the knee of the vitamin D-deficient swine. Therefore, we planned to evaluate the effect of vitamin D status (deficient, sufficient, and supplemented) on inflammation, TREMs, adiponectin, leptin, and change in the histology of the knee joint tissues in these microswine. We hypothesized that vitamin D supplementation should decrease inflammation in the knee joint tissue. The purpose of this study was to evaluate the beneficial immunomodulatory and anti-inflammatory roles of vitamin D in attenuating inflammation in the knee.

## Methods

### Porcine model

This study was conducted using Yucatan female microswine [36, 37]. In this study, 11 microswine were fed a pelleted, high-cholesterol diet (Teklad Miniswine Diet; Envigo, Indianapolis, IN, USA) with 17.4 % protein, 45.2 % carbohydrate, and 10.0 % fat by weight. Of the total kilocalories derived from the diet, 20.4 % of the kilocalories were from protein, 53.1 % were from carbohydrate, and 26.4 % were from fat. Two swine were fed a pelleted, high-fructose diet (1.5 IU/g vitamin D, 17.6 % fructose, 4 % cholesterol, 1.5 % sodium cholate; Teklad Miniswine Diet TD-150253) with 17.4 % protein, 46.8 % carbohydrate, and 10.0 % fat by weight. Of the total kilocalories derived from the diet, 20.1 % of the kilocalories were from protein, 54.0 % were from carbohydrate, and 26.0 % were from fat. The pelleted diet was either vitamin D-deficient (TD-150251), vitamin D-sufficient (TD-150250; 1.5 IU/g vitamin D, 4.5 % hydrogenated vegetable oil, 4 % cholesterol, 1.5 % sodium cholate) with 1500 IU/day of vitamin D_3_, or supplemented (TD-150252; 5 IU/g vitamin D, 4.5 % hydrogenated vegetable oil, 4 % cholesterol, 1.5 % sodium cholate) with 5000 IU/day of vitamin D_3_. On the basis of our previous experience and other studies, vitamin D_3_ supplementation with 1500 IU/day for the vitamin D-sufficient group or 5000 IU/day for the vitamin D-supplemented group was added to achieve normal (21–29 ng/ml) or supplemented serum levels of vitamin D in microswine [33, 38, 39]. Serum 25(OH)D levels were measured regularly. Blood serum levels of vitamin D were measured and recorded. The swine were placed into three respective categories based upon their 25(OH)D levels. The 25(OH)D-level parameters used for the classification were as follows: Levels in vitamin D-deficient (VDDef) swine were ≤20 ng/ml; levels in vitamin D-sufficient (VDSuff) swine were 30–44 ng/ml; and levels in vitamin D-supplemented (VDSupp) swine were >44 ng/ml. Five swine were in the VDDef group, 5 were in the VDSuff group, and 3 were in the VDSupp group, for a total of 13 swine in this study. After 1 year of being fed the diet, the microswine were killed, and knee joint tissues were collected.

### Tissue acquisition

The knee joint tissues (suprapatellar fat, quadriceps muscle, articular cartilage of tibial tuberosity, patellar tendon, collateral ligaments, medial and lateral menisci, infrapatellar fat pad, and synovial membrane) were harvested quickly after the swine were killed. Then the tissues were transported to a laboratory and fixed in 4 % formalin buffer for 24 h. Each specimen was transversely sectioned at 2 mm, processed in a Tissue-Tek VIP tissue processor (Sakura Finetek, Torrance, CA, USA), and embedded in paraffin. Thin sections (5 μm) were cut using a microtome (Leica Biosystems, Buffalo Grove, IL, USA) and subsequently placed on slides for hematoxylin and eosin (H&E) staining and immunofluorescence evaluation.

### Hematoxylin and eosin staining of specimens

All the knee joint tissues were stained with H&E following the manufacturer’s standard protocol (Newcomer Supply, Middleton, WI, USA). Stained slides were analyzed for the presence or absence of inflammation and fatty infiltration. All the slides were reviewed by a board-certified pathologist. All the images were scanned at × 20 magnification using an Olympus BX51inverted microscope (Olympus Scientific Solutions, Waltham, MA, USA) with a scale bar of 200 μm. The average adipocyte size in infra- and suprapatellar fat was calculated by selecting 15 random adipocytes in the different swine groups in each category using ImageJ software (National Institutes of Health [NIH], Bethesda, MD, USA). Chondrocyte counting was done using three high-power field images, and the average number of clustered chondrocytes to total chondrocytes per high-power field in each group was calculated. Areas of fatty infiltration were scanned, and fatty infiltration within the matrix of muscle, tendon, and ligament was calculated by measuring the area of all adipocytes present in the matrix followed by calculating the percentage of infiltration area per field.

### Histological analysis for proteoglycans, collagens, and matrix metalloproteinases

Tissue sections were stained with Alcian blue and Safranin-O Fast Green staining for analysis of the cartilage mucosubstance content in VDDef, VDSuff, and VDSupp swine cartilage. The stained slides were scanned by using an Olympus microscope at × 40 magnification. The results of the stained slides were graded in a blinded manner by a board-certified pathologist on a scale of 0–5 where 0 = minimal blue staining, 1 = very weak blue staining, 2 = weak blue staining, 3 = moderate blue staining, 4 = strong blue staining, and 5 = very strong blue staining for Alcian blue, and on a scale of 0–5 where 0 = minimal orange-red staining, 1 = very weak orange-red staining, 2 = weak orange-red staining, 3 = moderate orange-red staining, 4 = strong orange-red staining, and 5 = very strong orange-red staining for Safranin-O Fast Green staining [40].

Additionally, the tissue sections were examined by modified Russell-Movat pentachrome staining to assess the collagen and mucosubstance content of cartilage in VDDef, VDSuff, and VDSupp swine. The slides were double-checked by the pathologist in a blinded manner. The stained slides were scanned with an Olympus microscope at × 20 magnification and graded on a scale of 0–5 for collagen (yellow staining) and mucinous substance (blue-green staining), where 0 = minimal yellow/blue-green staining, 1 = very weak yellow/blue-green staining, 2 = weak yellow/blue-green staining, 3 = moderate yellow/blue-green staining, 4 = strong yellow/blue-green staining, and 5 = very strong yellow/blue-green staining.

The tissue sections were also subjected to immunohistochemical (IHC) staining for aggrecans and proteoglycans (aggrecans, decorins, and versicans). The IHC staining was conducted as per the standard protocol in our laboratory. Antigoat aggrecan (sc-25674; Santa Cruz Biotechnology, Dallas, TX, USA) for aggrecans in a 1:50 dilution and antimouse decorin (6D6-c; Developmental Studies Hybridoma Bank, Iowa City, IA, USA) and antimouse versican (12C5-c; Developmental Studies Hybridoma Bank) antibodies in a 1:100 dilution were used as the primary antibodies. A biotinylated secondary antibody was used, and diaminobenzidine was used as a fluorochrome to develop the slides. The slides were counterstained with hematoxylin to stain the nuclei. The slides were blindly checked by the pathologist. The slides were scanned at × 40 magnification with an Olympus microscope, and the total number of immunopositive cells for aggrecans, decorins, and versicans were counted on three different images for all tissues using ImageJ software. Cell density per square millimeter was calculated in the VDDef, VDSuff, and VDSupp groups.

### Immunofluorescence studies

Deparaffinization, rehydration, and antigen retrieval were performed prior to immunostaining. Immunofluorescent staining was done as per the standard protocol in our laboratory. As primary antibodies, we used rabbit anti-TREM-1 (PAA213Po01; Cloud-Clone Corp., Houston, TX, USA), rabbit anti-TREM-2 (bs-2723R; Bioss Inc., Woburn, MA, USA), and mouse anti-CD14 (ab182032; Abcam, Cambridge, MA, USA) for macrophages, mouse antiadiponectin (ab22554; Abcam), and rabbit antileptin (ab16227; Abcam) at 1:200 dilution, as well as mouse anti-neutrophil elastase (sc-53388; Santa Cruz Biotechnology) at 1:50 dilution. Alexa Fluor 594-conjugated (red) and Alexa Fluor 488-conjugated (green) secondary antibodies (Life Technologies, Carlsbad, CA, USA) at 1:500 dilution were used. The slides were counterstained with 4′,6-diamidino-2-phenylindole (DAPI) to stain nuclei. Negative controls were run by using an isotype antibody for each fluorochrome. Immunofluorescence microscopy was carried out with an Olympus BX51 inverted fluorescence microscope. The images were scanned at × 20 magnification and were reviewed by a board-certified pathologist. The mean fluorescence intensity (MFI) for TREM-1, TREM-2, adiponectin, and leptin was quantified by using ImageJ software. Two images from each swine in each group were taken for the measurement of MFI. The number of TREM-2 and CD-14 dual-positive cells was counted in five high-power fields for each tissue in all swine, and the average number of TREM-2 and CD-14 dual-positive cells per square millimeter was calculated for each group.

The analysis for the collagen types I, II, and III content in the VDDef, VDSuff, and VDSupp swine was carried out by immunofluorescent staining. Antirabbit collagen I (sc-30136; Santa Cruz Biotechnology), antigoat collagen II (sc-389924; Santa Cruz Biotechnology), and antirabbit collagen III (sc-28888; Santa Cruz Biotechnology) antibodies in 1:50 dilution were used as primary antibodies. Alexa Fluor 594-conjugated (red) and Alexa Fluor 488-conjugated (green) secondary antibodies (Life Technologies) at 1:500 dilution were used. DAPI was used to stain the nuclei. The stained slides were blindly double-checked by the pathologist. MFI for collagen II was calculated using three separate images for all swine in each group. ImageJ software was used to analyze the MFI. Average MFI was calculated for the three different groups of swine.

The analysis for the matrix metalloproteinases (MMPs) in the VDDef, VDSuff, and VDsupp swine was also carried out by immunofluorescent staining. Antigoat MMP-1 (sc-21731; Santa Cruz Biotechnology), antimouse MMP-2 (sc-53630; Santa Cruz Biotechnology), antimouse MMP-8 (sc-101450; Santa Cruz Biotechnology), antigoat MMP-9 (sc-6840; Santa Cruz Biotechnology), and antimouse MMP-13 (sc-101564; Santa Cruz Biotechnology) antibodies in a 1:50 dilution were used as primary antibodies. Alexa Fluor 594-conjugated (red) and Alexa Fluor 488-conjugated (green) secondary antibodies (Life Technologies) at 1:500 dilution were used. DAPI was used to stain the nuclei. The stained slides were blindly double-checked by the pathologist. MFI for MMP-9 was calculated using the three separate images for all swine in each group. ImageJ software was used to analyze the MFI. Average MFI was calculated for the three different groups of swine.

### Analysis of the vitamin D status with TREM-2 MFI, macrophage density, chondrocyte density and clustering, and adipocyte area

To correlate vitamin D status with various parameters of interest, correlation analysis was performed. The average values for the macrophage density in the VDDef, VDSuff, and VDSupp groups were converted into logarithm values to fit them in the range of values for other parameters (to fit them into a graph).

### Statistical analysis

Results are presented as mean ± SD for each parameter. The Wilcoxon two-samples test was used for statistical analysis of the significance of differences between the VDDef, VDSuff, and VDSupp groups. A *p* value <0.05 was considered significant.

## Results

### Biochemical parameters

The serum levels of vitamin D at the beginning of the study were <15 ng/ml in the VDDef group, 17.53 ± 1.13 ng/ml in the VDSuff group, and 31.93 ± 3.04 ng/ml in the VDSupp group. The experimental diets were started when the swine were at the age of 6 months. After 1 year of feeding the swine the experimental diet, the mean serum 25(OH)D levels were 8.1 ± 1.13 ng/ml in the VDDef group, 24.01 ± 0.72 ng/ml in the VDSuff group, and 52.58 ± 5.68 ng/ml in the VDSupp group. The corresponding average levels of serum parathyroid hormone were 38.57 ± 5.75 ng/ml, 13.03 ± 3.23 ng/ml, and 8.52 ± 2.19 ng/ml, respectively; serum calcium, 8.60 ± 0.78 ng/ml, 10.02 ± 0.22 ng/ml, and 9.7 ± 0.05 ng/ml, respectively; and serum cholesterol, 512.01 ± 56.9 ng/ml, 551.75 ± 45.03 ng/ml, and 461 ± 43.08 ng/ml, respectively. The corresponding average weights at the time the animals were killed were 95.96 ± 4.53 lb, 112.24 ± 6.23 lb, 128.11 ± 13.97 lb, respectively (see Additional file 1).

### Increased inflammation in vitamin D-deficient group

**Fig. 1:**
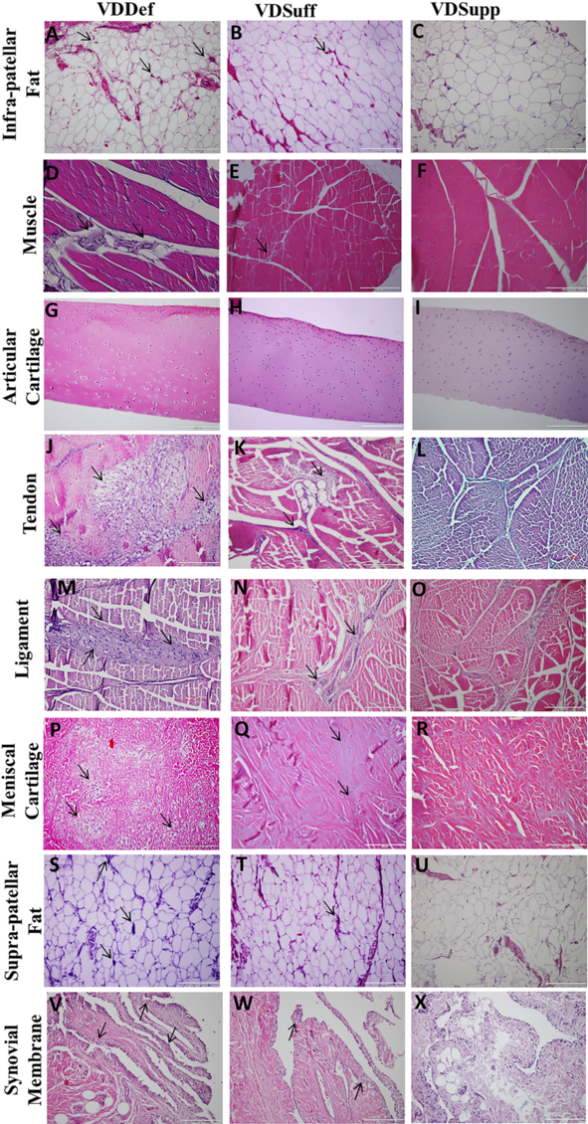
Hematoxylin and eosin (H&E) staining of infrapatellar fat, quadriceps muscle, articular cartilage, patellar tendon, collateral ligament, meniscal cartilage, suprapatellar fat, and synovial membrane. H&E staining shows the histology and inflammation in the vitamin D-deficient (VDDef), vitamin D-sufficient (VDSuff), and vitamin D-supplemented (VDSupp) group infrapatellar fat (**a**, **b**, **c**), muscle (**d**, **e**, **f**), articular cartilage (**g**, **h**, **i**), tendon (**j**, **k**, **l**), ligament (**m**, **n**, **o**), menisci (**p**, **q**, **r**), suprapatellar fat (**s**, **t**, **u**), and synovial membrane (**v**, **w**, **x**). *Arrows* indicate the presence of inflammatory cells in the tissue. These are the representative images of five swine each in the VDDef and VDSuff groups and three swine in the VDSupp group

Microscopic analysis of H&E-stained sections of infrapatellar fat (Fig. 1a, b, c), quadriceps muscle (Fig. 1d, e, f), patellar tendon (Fig. 1j, k, l), collateral ligament (Fig. 1m, n, o), meniscal cartilage (Fig. 1p, q, r), suprapatellar fat (Fig. 1s, t, u), and synovial membrane (Fig. 1v, w, x) revealed increased inflammation in the VDDef group compared with the VDSuff and VDSupp groups. The inflammation in the VDSuff group was higher than that in the VDSupp group. In synovial membrane, there was mild inflammation and mild hyperplasia in the VDDef group, while the VDSuff and VDSupp groups showed minimal changes. Inflammation was defined on the basis of the presence of macrophages and/or neutrophils in the tissue matrix. Knee joint tissues in the VDDef group showed the presence of many inflammatory cells along with the cluster of inflammation (>4 inflammatory cells), while there were only occasional or few inflammatory cells in the VDSuff and VDSupp groups (see Additional file 2).

### Increased fatty infiltration in vitamin D-deficient group

**Fig. 2:**
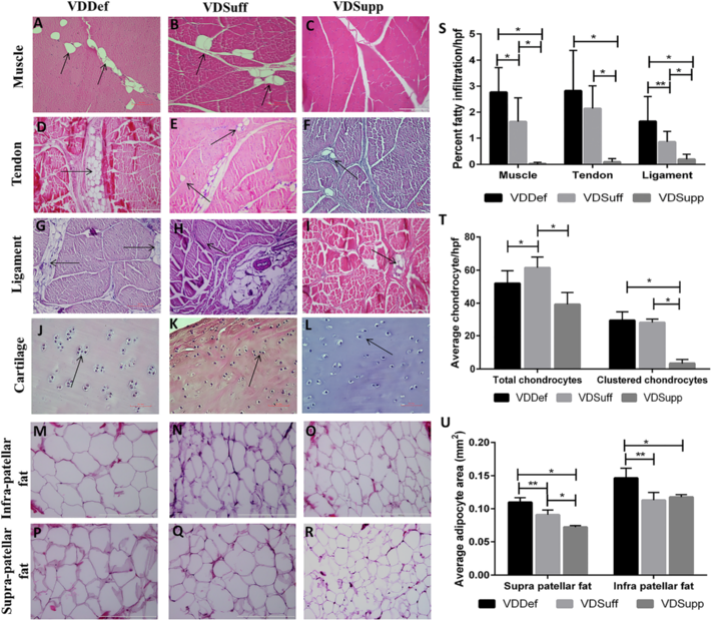
Hematoxylin and eosin (H&E) staining of quadriceps muscle, patellar tendon, collateral ligaments, articular cartilage, and infra- and suprapatellar fat pads. H&E staining shows the fatty infiltration in the vitamin D-deficient (VDDef), vitamin D-sufficient (VDSuff), and vitamin D-supplemented (VDSupp) groups: quadriceps muscle (**a**, **b**, **c**), patellar tendon (**d**, **e**, **f**), and collateral ligaments (**g**, **h**, **i**). Chondrocyte clustering (×40 original magnification) in the VDDef and VDSuff groups compared with the VDSupp group (**j**, **k**, **l**), as well as the adipocyte area in infrapatellar (**m**, **n**, **o**) and suprapatellar fat pads (**p**, **q**, **r**), is shown. The percentage of fatty infiltration per high-power field (**s**), average total and clustered chondrocytes (**t**), and average adipocyte area (**u**) are graphed. *Arrows* indicate the presence of adipocytes within the matrix of the tissue and chondrocyte clustering. These are the representative images of five swine each in the VDDef and VDSuff groups and three swine in the VDSupp group. Data are shown as mean ± SD. *p < 0.05, **p < 0.01

Histological analysis of H&E-stained sections of muscle, tendon, and ligament showed increased fatty infiltration in the VDDef group (Fig. 2a, d, g) compared with the VDSuff (Fig. 2b, e, h) and VDSupp (Fig. 2c, f, i) groups. There was minimal fatty infiltration in the VDSuff group, and no fatty infiltration was observed in the VDSupp group (see Additional file 2). Fatty infiltration was defined as the presence of adipocytes between muscle, tendon, and ligament fibers (i.e., not simply next to the structure, but interspersed within the structure). The percentage of fatty infiltration per field was calculated in the VDDef, VDSuff, and VDSupp groups (Fig. 2s). The presence of adipocytes interspersed within the structure was further confirmed by Oil Red O staining and immunopositivity for adiponectin around the adipocyte (data not shown).

### Increased chondrocyte clustering in vitamin D-deficient group

**Fig. 3:**
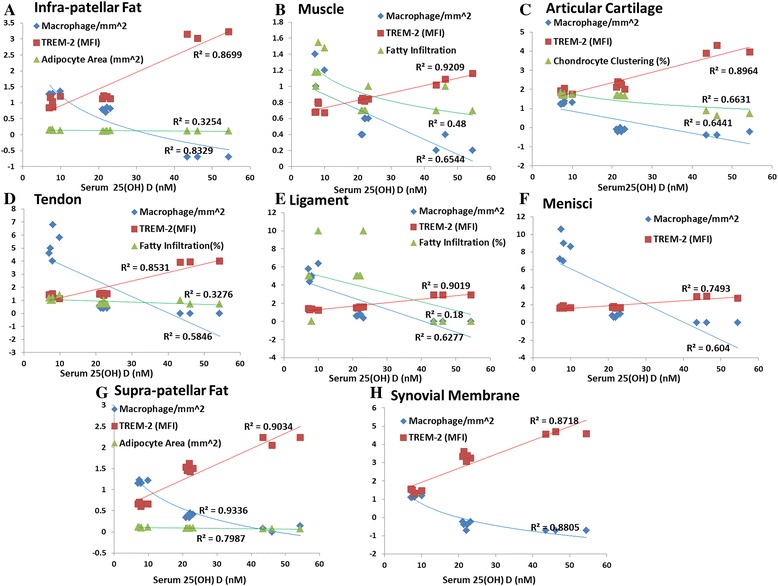
Analysis of changes in inflammation, fatty infiltration, adipocyte area, and chondrocyte clustering by vitamin D status in knee joint tissues. **a** Macrophage density (per square millimeter, logarithm values), triggering receptor expressed on myeloid cells (TREM)-2 mean fluorescence intensity (MFI), and adipocyte area (per square millimeter) in infrapatellar fat. **b** Macrophage density (per square millimeter), TREM-2 MFI, and fatty infiltration (percentage of area, logarithm values) in muscle. **c** Macrophage density (per square millimeter, logarithm values), TREM-2 MFI, and chondrocyte clustering (clustered/total chondrocytes, logarithm values) in articular cartilage. **d** Macrophage density (per square millimeter), TREM-2 MFI, and fatty infiltration (percentage of area, logarithm values) in tendon. **e** Macrophage density (per square millimeter), TREM-2 MFI, and fatty infiltration (percentage of area) in ligament. **f** Macrophage density (per square millimeter) and TREM-2 MFI in menisci. **g** Macrophage density (per square millimeter, logarithm values), TREM-2 MFI, and adipocyte area (per square millimeter) in suprapatellar fat. **h** Macrophage density (per square millimeter, logarithm values) and TREM-2 MFI in synovial membrane

Histological analysis of articular cartilage in the VDDef, VDSuff, and VDSupp groups revealed increased chondrocyte clustering in the VDDef group compared with the VDSuff and VDSupp groups (Fig. 2j, k) as well as in the VDSuff group (Fig. 2k) compared with the VDSupp group (Fig. 2l). Quantitation of chondrocytes per high-power field showed that nearly 50 % of chondrocytes were clustered in the VDDef and VDSuff groups, whereas only 3–4 % were clustered in the VDSupp group (Figs. 2t and 3c) (see Additional file 1).

### Increased adipocyte size in vitamin D-deficient group

Analysis of the average adipocyte area in suprapatellar (Fig. 2m, n, o) and infrapatellar (Fig. 2p, q, r) fat pads revealed significantly increased adipocyte size in the VDDef group compared with the VDSuff and VDSupp groups, as well as in the VDSuff group compared with the VDSupp group (Fig. 2u).

### Decreased expression of proteoglycans in vitamin D-deficient group

**Fig. 4:**
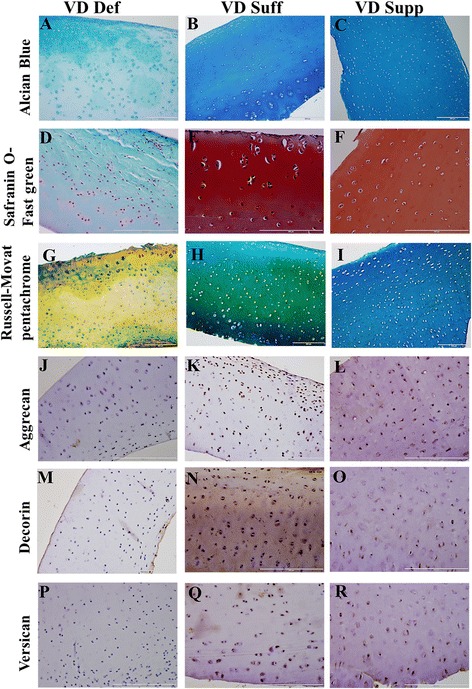
Alcian blue, Safranin-O Fast Green, modified Russell-Movat pentachrome, and immunohistochemical staining for matrix substance and proteoglycans (aggrecans, decorins, and versicans). Alcian blue (**a**, **b**, **c**), Safranin-O Fast Green (**d**, **e**, **f**), modified Russel-Movat pentachrome (**g**, **h**, **i**), aggrecans (**j**, **k**, **l**), decorins (**m**, **n**, **o**), and versicans (**p**, **q**, **r**) in the vitamin D-deficient (VDDef), vitamin D-sufficient (VDSuff), and vitamin D-supplemented (VDSupp) groups of swine. These are the representative images of five swine each in the VDDef and VDSuff groups and three swine in the VDSupp group

Analysis of Alcian blue, Safranin-O Fast Green, and modified Russell-Movat pentachrome staining and immunohistochemistry for proteoglycans (aggrecans, decorins, and versicans) showed decreased expression of matrix substance and proteoglycans in the VDDef group (Fig. 4a, d, g, j, m, p) compared with the VDSuff (Fig. 4b, f, h k, n and q) and VDSupp (Fig. 4c, f, i, l, o, r) groups. Comparison of the VDSuff group with the VDSupp group revealed increased expression of proteoglycans in the VDSuff group compared with the VDSupp group (Fig. 4). Proteoglycan and matrix substance expression in the VDSuff group swine was nonuniform, whereas the expression in the VDSupp group was uniform (Fig. 4, Table 1).

**Table 1:** Histological analysis of matrix substance and proteoglycans (aggrecan, decorin, and versican) for collagen loss in knee cartilage of VDDef, VDSuff, and VDSupp swine

Swine group	Alcian blue staining	Safranin-O and Fast Green staining	Modified Russell-Movat pentachrome staining (blue-green/yellow)	Aggrecan^+^ cells/mm^2^	Decorin^+^ cells/mm^2^	Versican^+^ cells/mm^2^
VDDef						
1	3	0	3/4	4.72 ± 0.84	12.16 ± 1.39	5.06 ± 1.23
2	1	0	2/4	0	0	0
3	2	0	3/3	0	0	0
4	2	2	3/4	18.51 ± 4.42	11.29 ± 0.41	10.4 ± 1.25
5	2	3	3/4	20.58 ± 2.85	11.16 ± 1.69	8.34 ± 0.52
VDSuff						
1	5	2	2/4	31.95 ± 4.23	56.22 ± 2.88	28.11 ± 1.90
2	2	2	4/2	20.18 ± 2.88	17.54 ± 3.01	13.38 ± 1.51
3	2	3	3/3	18.5 ± 1.5	18.97 ± 3.56	17.98 ± 1.20
4	2	5	3/1	51.18 ± 2.59	19.46 ± 3.3	20.90 ± 3.14
5	5	3	4/1	63.79 ± 8.19	29.85 ± 2.27	15.37 ± 1.5
VDSupp						
1	4	3	5/1	36.95 ± 3.48	15.78 ± 0.67	19.26 ± 1.69
2	4	4	5/1	27.54 ± 1.87	10.57 ± 2.2	9.61 ± 2.31
3	4	4	5/2	28.74 ± 1.88	11.05 ± 1.1	8.41 ± 1.81

*Abbreviations: VDDef* Vitamin D-deficient, *VDSuff* Vitamin D-sufficient, *VDSupp* Vitamin D-supplemented

Alcian blue, Safranin-O Fast Green, and modified Russell-Movat pentachrome staining were done for assessment of cartilage mucosubstance damage. Immunohistochemical staining was done for aggrecans, decorins, and versicans, and the immunopositive cell density was calculated. Data are presented as mean ± SD for immunopositive cell density. See the Methods section for discussion of cartilage grading

### Increased leptin and decreased adiponectin expression in vitamin D-deficient swine

**Fig. 5:**
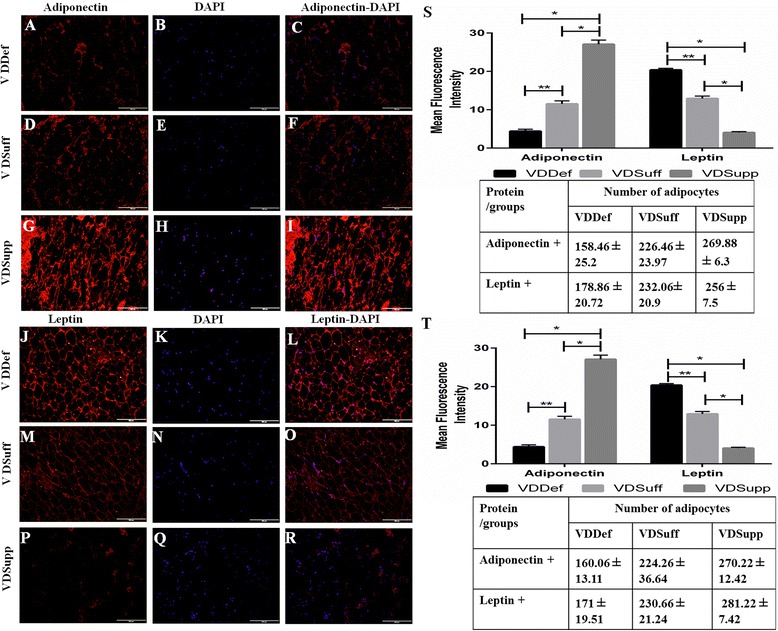
Immunofluorescent staining for adiponectin and leptin in infra- and suprapatellar fat pads. Immunofluorescent staining in the vitamin D-deficient (VDDef), vitamin D-sufficient (VDSuff), and vitamin D-supplemented (VDSupp) group infrapatellar fat is shown for adiponectin (**a**, **d**, **g**), leptin (**j**, **m**, **p**), 4′,6-diamidino-2-phenylindole (DAPI) (**b**, **e**, **h**, **k**, **n**, **q**), merged adiponectin-DAPI (**c**, **f**, **i**), and merged leptin-DAPI (**l**, **o**, **r**). The mean fluorescence intensity (MFI) of adiponectin and leptin with average adipocyte count (**s**) in infrapatellar fat, and the MFI of adiponectin and leptin with average adipocyte count in suprapatellar fat (**t**), is shown for VDDef, VDSuff, and VDSupp swine. These are the representative images of five swine each in the VDDef and VDSuff groups and three swine in the VDSupp group. Data are shown as mean ± SD. *p < 0.05, **p < 0.01

Immunofluorescence studies of infrapatellar and suprapatellar fat revealed greater immunoreactivity for leptin and lesser immunoreactivity for adiponectin in the VDDef group (Fig. 5a, j; Additional file 3a, j) than in the VDSuff (Fig. 5d, m; Additional file 3d, m) and VDSupp (Fig. 5g, p; Additional file 3 g, p) groups. The MFI of leptin was higher and that of adiponectin was lower in the VDDef group than in the VDSuff and VDSupp groups (Fig. 5s, t). The average adipocyte counts in infra- and suprapatellar fat pads were higher in the VDDef group than in the VDSuff and VDSupp groups, and they were higher in the VDSuff group than in the VDSupp group (Fig. 5s, t).

### Increased expression of TREM-2 in vitamin D-sufficient and vitamin D-supplemented groups

**Fig. 6:**
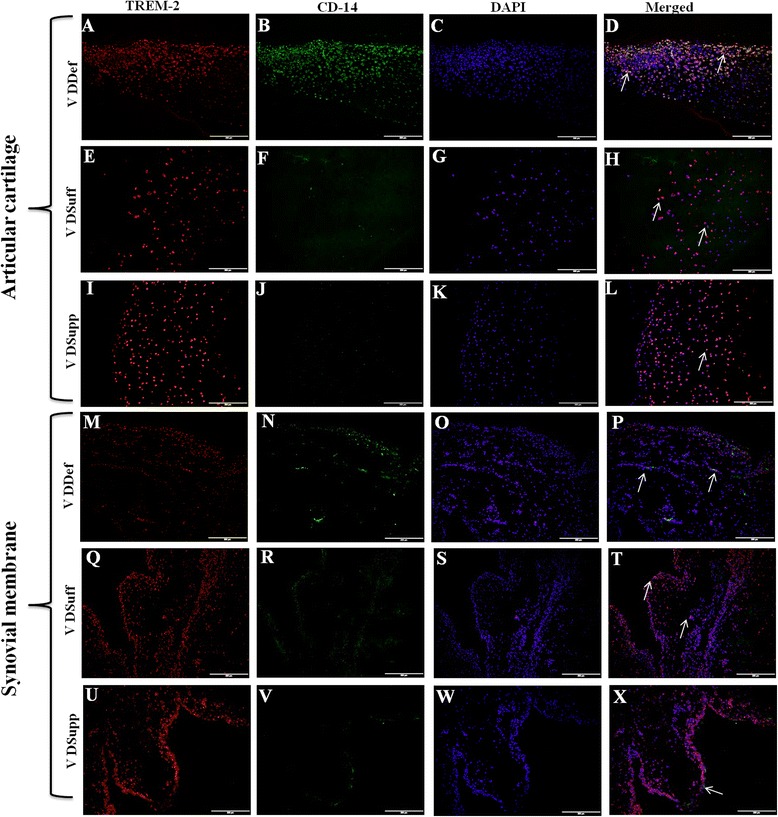
Immunofluorescent staining for triggering receptor expressed on myeloid cells (TREM)-2 and macrophage (CD14) as well as colocalization of TREM-2 with CD14^+^ cells in articular cartilage and synovial membrane. Immunofluorescent staining for TREM-2 (**a**, **e**, **i**, **m**, **q**, **u**), CD14 (**b**, **f**, **j**, **n**, **r**, **v**), 4′,6-diamidino-2-phenylindole (DAPI) (**c**, **g**, **k**, **o**, **s**, **w**), and merged images for examining colocalization of TREM-2 and CD14 (**d**, **h**, **l**, **p**, **t**, **x**). *Arrows* indicate colocalization of TREM-2 with CD14. These are the representative images of five swine each in the vitamin D-deficient (VDDef) and vitamin D-sufficient (VDSuff) groups and three swine in the vitamin D-supplemented (VDSupp) group

**Fig. 7:**
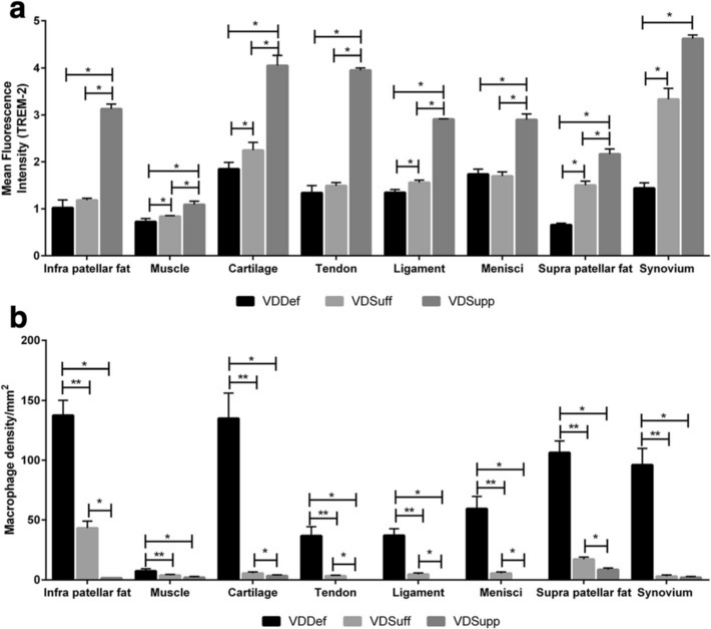
Analysis of triggering receptor expressed on myeloid cells (TREM)-2 immunoreactivity and macrophage density. Mean fluorescence intensity of TREM-2 (**a**) and number of macrophages (CD14^+^) (**b**) in the vitamin D-deficient (VDDef) (*n* = 5), vitamin D-sufficient (VDSuff) (*n* = 5), and vitamin D-supplemented (VDSupp) (*n* = 3) group infrapatellar fat, muscle, articular cartilage, tendon, ligament, menisci, suprapatellar fat, and synovial membrane. A comparative analysis was performed between the VDDef, VDSuff, and VDSupp groups. Data are shown as mean ± SD. *p < 0.05, **p < 0.01

Immunofluorescence studies of articular cartilage (Fig. 6a, e, i), synovial membrane (Fig. 6a, q, u), infrapatellar fat (see Additional file 4a, e, i), quadriceps muscle (see Additional file 4 m, q, u), patellar tendon (see Additional file 5a, e, i), collateral ligaments (see Additional file 5m, q, u), meniscal cartilage (see Additional file 6a, e, i), and suprapatellar fat (see Additional file 6 m, q, u) revealed higher immunoreactivity for TREM-2 in the VDSupp group than in the VDSuff and VDDef groups and in the VDSuff group than in the VDDef group (Fig. 3). The MFI of TREM-2 was significantly higher (Fig. 7a) in the VDSuff and VDSupp groups than in the VDDef group. The colocalization of TREM-2 with macrophages was observed more in the VDDef group than in the VDSuff and VDSupp groups and in the VDSuff group than in the VDSupp group. We found very minimal positivity for TREM-1 in articular cartilage, tendon, ligament, and suprapatellar fat (see Additional file 7a, e, i, m).

### Decreased expression of collagen II and increased expression of MMP-9 in vitamin D-deficient group

**Fig. 8:**
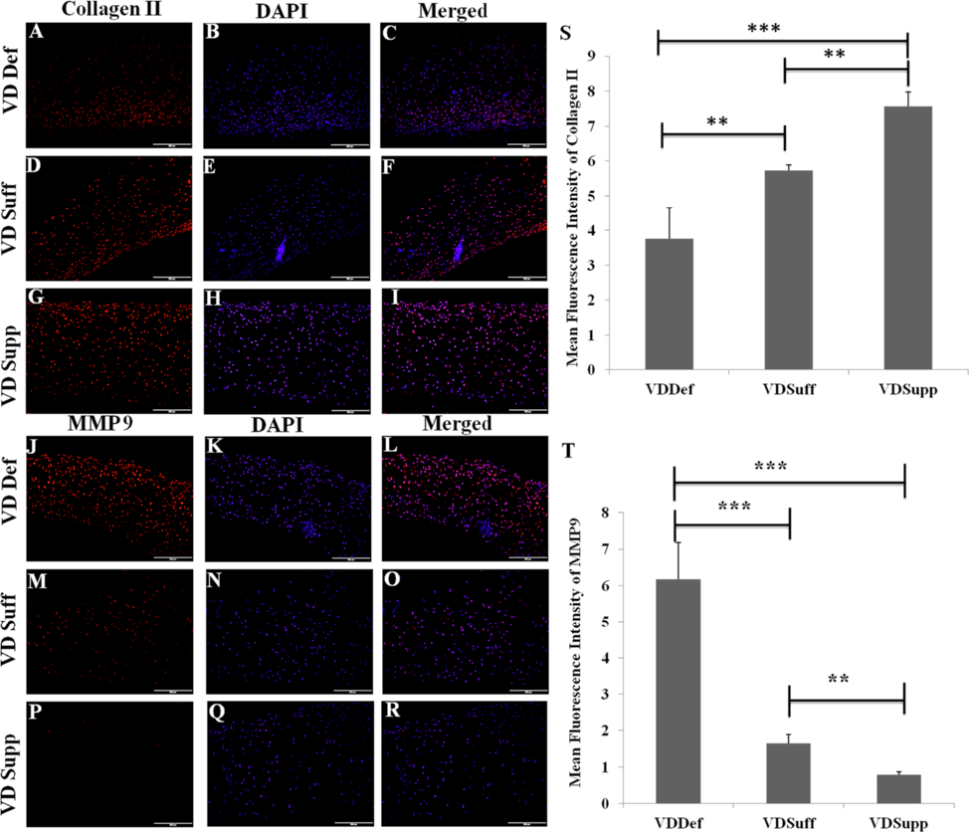
Immunofluorescence studies for collagen type II and matrix metalloproteinase 9 (MMP-9) in cartilage. Immunofluorescent staining is shown for collagen II (**a**, **d**, **g**), MMP-9 (**j**, **m**, **p**), 4′,6-diamidino-2-phenylindole (DAPI) (**b**, **e**, **h**, **k**, **n**, **q**), and merged images (**c**, **f**, **i**, **l**, **o**, **r**). Mean fluorescence intensity is shown for collagen II (**s**) and MMP-9 (**t**). These are the representative images of five swine each in the vitamin D-deficient (VDDef) and vitamin D-sufficient (VDSuff) groups and three swine in the vitamin D-supplemented (VDSupp) group. Data are shown as mean ± SD. **p < 0.01, ***p < 0.001

Immunofluorescent staining for collagen I, II, and III revealed higher immunoreactivity of collagen II than for collagen I and III in all cartilage tissues (see Additional file 8a, d, g). The immunofluorescence studies for collagen II revealed decreased immunoreactivity in the VDDef group compared with the VDSuff and VDSupp groups (Fig. 8a, d, g), and in the VDSuff group compared with the VDSupp group (Fig. 8d, g). The immunofluorescence studies showed no immunoreactivity for MMP-1, MMP-2, and MMP-13 (see Additional file 9a, d, m), very minimal immunoreactivity for MMP-8 (see Additional file 9g), and higher immunoreactivity for MMP-9 (see Additional file 9j) in the VDDef, VDSuff, and VDSupp groups. The immunoreactivity of MMP-9 was higher in the VDDef group than in the VDSuff and VDSupp groups (Fig. 8j, m, p) and in the VDSuff group than in the VDSupp group (Fig. 8m, p). The MFI of collagen II (Fig. 8s) and MMP-9 (Fig. 8t) was significantly higher in the VDDef group than in the VDSuff and VDSupp groups and in the VDSuff group than in the VDSupp group.

### Increased number of macrophages in vitamin D-deficient group

Dual-immunofluorescence studies of TREM-2 and macrophage for suprapatellar and infrapatellar fat, muscle, tendon, ligament, meniscal cartilage, articular cartilage, and synovial membrane showed significantly an increased number of macrophages in the VDDef group compared with the VDSuff and VDSupp groups (Fig. 3). Higher numbers of CD14^+^ cells were found colocalized with TREM-2 in the VDDef group than in the VDSuff and VDSupp groups (Fig. 7b). Fewer macrophages colocalized with TREM-1 in articular cartilage, tendon, ligament, and suprapatellar fat in the VDDef group were also observed (see Additional file 7). Dual-immunofluorescence of TREM-1, TREM-2, and neutrophils showed no positivity for neutrophil elastase, suggesting the absence of neutrophils in the knee joint tissues (data not shown).

## Discussion

On the basis of microscopic analysis of H&E staining, compared with the VDSupp group, in the VDDef and VDSuff groups we found increased inflammation in knee joint tissues; increased fatty infiltration in quadriceps muscle, patellar tendon, and collateral ligaments; and increased chondrocyte clustering. These findings were further supported by immunofluorescence studies. Histological analysis of the matrix substance, proteoglycans, and collagens showed increased cartilage matrix loss in the VDDef group compared with the VDSuff and VDSupp groups and in the VDSuff group compared with the VDSupp group. There was increased expression of MMP-9 in the VDDef group compared with the VDSuff and VDSupp groups and in the VDSuff group compared with the VDSupp group. The very weak light blue staining in VDDef group compared with VDSuff and VDSupp group, as well as the moderate blue staining in the VDSuff group compared with very strong blue staining in the VDSupp group by Alcian blue staining, suggests loss of sulfated mucosubstances of the cartilage with decreasing vitamin D. Similarly, absence of orange to red coloring in the VDDef group and presence of orange-red coloring in VDSuff and VDSupp group suggest loss of cartilage and mucinous matrix substance. In modified Russell-Movat pentachrome staining, the VDDef group showed more yellow coloration than the VDSuff and VDSupp groups, while the VDSuff group showed blue-green coloration and the VDSupp group had strong blue coloration. The predominance of yellow coloration in VDDef group may be due to the loss of matrix mucosubstance and exposure of the collagen fibrils, whereas in the VDSuff and VDSupp groups, strong blue coloration indicating the presence of matrix mucosubstance may have masked the yellow coloration of the collagen fibers, as they were not exposed. These results suggest the beneficial role of vitamin D supplementation in protecting the cartilage matrix by increasing matrix substance and proteoglycans.

Inflammation and fatty infiltration may render the muscle weak and contribute to OA [2, 6]. The architectural changes or distortion of the normal architecture in quadriceps muscles, patellar tendon, and collateral ligaments with the foci of inflammation and fatty infiltration suggest that inflammation and fatty infiltration can change the knee joint morphology in vitamin D deficiency. Absence of architectural changes and fatty infiltration in the VDSupp group suggests the potential beneficial role of vitamin D in decreasing inflammation and fatty infiltration in knee joint tissues. These results are in accordance with those of previous studies suggesting a restorative effect on muscle weakness with vitamin D supplementation [6]. Distortion of muscle structure may lead to muscle weakness, and it has been suggested that muscle weakness is an early symptom of OA. Low levels of vitamin D are associated with muscle weakness and impaired function [41]. Muscle weakness results in decreased shock-absorbing capacity in knee joint and precedes and mediates cartilage degeneration [2, 42]. Our findings suggest a role for vitamin D supplementation in decreasing inflammation in tendon and ligament in addition to its beneficial effects on muscle.

Obesity has long been considered a risk factor for OA. Due to the extension of the metabolic effects of obesity to the fat depots in the knee joint, suprapatellar fat (SPF), and infrapatellar fat (IPF), inflammation in SPF and IPF may play a significant role in knee joint or patellofemoral joint OA [43, 44]. White adipocytes acting as endocrine cells secrete adipokines and cytokines that regulate various functions in the human body, including angiogenesis and the immune response [45]. Histological analysis of knee joint tissues revealed inflammation in IPF and SPF, mainly in VDDef swine compared with minimal inflammation in the VDSuff group and no inflammation in the VDSupp group. We further stained these tissues for the adipokines adiponectin and leptin. Immunofluorescence studies revealed lower levels of adiponectin and higher levels of leptin in the VDDef group than in the VDSuff and VDSupp groups. Adiponectin may play a protective role in the pathogenesis of OA by increasing expression of tissue inhibitors of metalloproteinases and decreasing MMPs, and it may possess several anti-inflammatory properties by suppressing nuclear factor kB, interleukin 6, and tumor necrosis factor α [7]. Adiponectin levels have been considered as a biomarker associated with early radiographic disease progression in early rheumatoid arthritis (RA) [46]. Further, increased adiponectin levels in synovial fluid are associated with RA and are thought to counteract the local inflammatory process [47]. Increased expression of adiponectin in the VDSupp group in this study may also suggest a role of vitamin D in decreasing inflammation and increasing production of adiponectin to protect the knee joint. Further, the role of vitamin D deficiency in other forms of inflammatory arthritis, such as RA, has been discussed, and vitamin D intake has been negatively correlated with the development of RA [48]. Increased levels of leptin are detrimental to chondrocytes and are associated with cartilage degeneration and volume loss [31, 32]. In accordance with these studies, we also found decreased adiponectin and increased leptin levels in the VDDef group compared with the VDSuff and VDSupp groups, suggesting a role of vitamin D in enhancing adiponectin and attenuating leptin levels, probably by decreasing inflammation.

Fatty infiltration of quadriceps muscle, patellar tendon, and collateral ligaments was associated with increased inflammation in areas of fatty infiltration in the VDDef and VDSuff groups. There was only minimal inflammation in the VDSupp group. Immunofluorescence studies of knee joint tissues for TREM-1 and TREM-2 showed minimal immunoreactivity for TREM-1, but, to our surprise, all the tissues in the VDDef, VDSuff, and VDSupp groups showed immunoreactivity for TREM-2, although TREM-2 immunoreactivity was higher in the VDSupp group than the VDDef and VDSuff groups. In agreement with the literature which suggests that TREM-1 expression increases during inflammation [27–29], in our study only few tissues showed TREM-1 immunoreactivity. The high TREM-2 immunoreactivity in the VDDef group in inflamed tissues was of interest, so we performed a dual-immunofluorescence study of TREM-1 and TREM-2 with macrophages and neutrophils. Dual-immunofluorescence revealed higher number and more colocalization of macrophages with TREM-2 in the VDDef group. This suggests that higher TREM-2 expression in the presence of macrophages may be due to the protective response or to innate immunity of the body. Because macrophage populations are highly heterogeneous, changes in heterogeneity of macrophages may play a role in OA. The lower immunoreactivity for TREM-1 and higher immunoreactivity for TREM-2 also suggest a predominance of M2 macrophages, the anti-inflammatory macrophages, compared with M1 macrophages, which are proinflammatory. The change in heterogeneity of the macrophage plays a role in bone remodeling or osteogenesis by changing the microenvironment [49], but the role of heterogeneity in the pathogenesis of OA needs to be determined. Further, the absence of neutrophils and the presence of macrophages only as the inflammatory cells suggest the chronicity of inflammation in the knee joint [50, 51] and a possible role of macrophages in the inflammatory response. Vitamin D may have a pleiotropic effect in immune cells, including macrophages, and an immunomodulatory role of vitamin D in various immune cells and diseases has been discussed [52]. The role of vitamin D in blocking the inflammatory cytokines, cell proliferation, and angiogenesis in the context of various diseases has been studied, and potential beneficial immunomodulatory and anti-inflammatory roles have been elucidated [53, 54]. In this study, the higher expression of TREM-2 in the VDSupp group suggests an anti-inflammatory role of vitamin D in knee joint tissues. The decreased colocalization of TREM-2 with macrophages in dual-immunofluorescence studies in the VDSupp group compared with the VDDef group further supports the anti-inflammatory role of vitamin D. These findings are in accordance with previous studies suggesting decreased vitamin D receptor expression during inflammation and an inflammation-attenuating effect of vitamin D supplementation [33, 55].

Loss of cartilage volume and subchondral bone is the primary pathophysiology of OA. Prevention of bone damage to reduce progression of cartilage damage and loss may reduce the severity and progression of OA [56], and vitamin D can play an important role in attenuating bone damage [14]. Clustering of chondrocytes in degenerating cartilage has been suggested as a hallmark of OA and classified as grade 2 cartilage degeneration by the International Cartilage Repair Society [57, 58]. Chondrocyte clustering is also a feature of autoimmune arthritis (i.e., RA), where chronic inflammation does play a role in the pathogenesis. Clustering of cells has also been discussed in other degenerative joint diseases involving intervertebral discs, menisci, and cricoarytenoid cartilage. Chondrocyte clustering may be due to the disease progression in OA or to the increased proliferation or migration of cells from the deeper layers [59]. In this study, we found chondrocyte clustering (50 % of the total chondrocytes) in the VDDef and VDSuff groups and only 3 % clustering in the VDSupp group. We also observed that chondrocyte clustering was more prominent toward the edges of cartilage. These data suggest that vitamin D supplementation may have led to the decreased clustering in the VDSupp group. As the chondrocyte clustering is associated with cartilage degeneration, vitamin D supplements may act as an intervening agent to decrease the progression of cartilage loss. It has also been suggested that clustered chondrocytes near degenerating cartilage have progenitor characteristics with proliferative potential [58]; vitamin D as a proliferative agent may thus act as an adjunctive therapy.

Matrix degeneration occurs as a result of increased levels of degrading enzymes, and the activity of these metalloproteinase enzymes is modulated by vitamin D. Low levels of vitamin D may lead to increased production of MMPs [60]. In this study, we found increased levels of MMP-9 expression in VDDef swine compared with VDSuff and VDSupp swine. Further, the MMP-9 levels were higher in VDSuff swine than in VDSupp swine. These results suggest that vitamin D supplementation attenuated the expression of MMP-9 in cartilage. The expression of collagen II was decreased in the VDDef group compared with the VDSuff and VDSupp groups and in the VDSuff group compared with the VDSupp group, which can be explained by the higher levels of MMP-9 in VDDef and VDSuff swine. Further, vitamin D deficiency is associated with smaller proteoglycan monomers [61], and our results showing decreased proteoglycans (aggrecans, decorins, and versicans) in VDDef swine support the loss of proteoglycans in that group. The increased expression of proteoglycans in the VDSuff and VDSupp groups suggests a beneficial role of vitamin D. There was uniformity of expression of matrix substance and proteoglycans in the VDSupp group, but the expression was nonuniform in VDSuff swine. The higher expression of matrix substance and proteoglycans in two swine in the VDSuff group can be explained by decreased aggrecan expression through its downregulation at the posttranscriptional level with vitamin D_3_ [62]. Vitamin D_3_ may also potentiate the MMP expression in activated chondrocytes, facilitating intrinsic chondrolysis in the presence of proinflammatory cytokines, and it may suppress the MMPs in activated synovial fibroblasts to decrease chondrolysis. Hence, vitamin D may not directly affect the MMPs and can modulate production of other cytokines involved in the process of chondrolysis [63]. Although serum vitamin D at baseline levels has an association with OA, a low vitamin D level did not predict the loss of articular cartilage or narrowing of the joint space [64]. However, a higher level of vitamin D has been associated with a higher rate of hospitalization for knee or hip joint OA [65]. Hence, there is a need to further research the role of vitamin D in the pathogenesis of OA.

### Limitations of the study

A limitation of this study was the absence of control domestic swine fed a normal diet. Another limitation may be the small sample size in the VDSupp group. Despite these limitations, the attenuation of inflammation and fatty infiltration in the VDSupp group by vitamin D provides a new basis for further research into the role of vitamin D in OA pathogenesis.

## Conclusions

Vitamin D supplementation is associated with attenuation of inflammation, fatty infiltration, chondrocyte clustering, and preservation of the tissue architecture. The histological and immunofluorescence results of the present study are closely related and thus validate the microscopic findings. The results of this study demonstrate the potential beneficial effect of vitamin D in decreasing inflammation and fatty infiltration in knee joints, which may decrease pain and disability. However, the role of vitamin D supplementation in decreasing the progression of cartilage loss or OA needs to be investigated.

## Additional files

Additional file 1: Table S2. Baseline table for swine groups. The study included a total of 13 swine: 5 in the VDDef group, 5 in the VDSuff group, and 3 in the VDSupp group. Weight is indicated in pounds (lb). Average vitamin D_3_ levels were measured after 1 year of vitamin D diet intervention. Chondrocyte clustering was measured at × 40 magnification. (DOCX 13 kb) Additional file 2: Table S3. Overview of all tissues with regard to inflammation and fatty infiltration. Inflammation in knee joint tissues (suprapatellar fat, infrapatellar fat, muscle, tendon, ligament, and menisci) was subjectively graded on the basis of the following criteria: nil = no inflammation; 1+ = occasional inflammatory cells; 2+ = few inflammatory cells; 3+ = many inflammatory cells; and 4+ = clumps or clusters of inflammatory cells. Macrophage density was calculated for inflammation in cartilage of all knee joints. Macrophage density (CD14^+^ cells) in all the knee joint tissues was analyzed for objective grading of inflammation (Fig. 6). Subjective grading of fatty infiltration was based on the following criteria: no fatty infiltration; minimal fatty infiltration = fatty infiltration in up to 5 % of tissue area; mild fatty infiltration = fatty infiltration in 6–25 % of tissue area; moderate fatty infiltration = fatty infiltration in 25–50 % of tissue area; and severe fatty infiltration = fatty infiltration in >50 % of tissue area. Further, the percentage of fatty infiltration was calculated (Fig. 2). (DOCX 16 kb) Additional file 3: Figure S9. Immunofluorescent staining for adiponectin and leptin in suprapatellar fat pad. Immunofluorescent staining in the VDDef, VDSuff, and VDSupp suprapatellar fat pads for adiponectin (**a**, **d**, and **g**), leptin (**j**, **m**, and **p**), DAPI (**b**, **e**, **h**, **k**, **n**, and **q**), merged adiponectin-DAPI (**c**, **f**, and **i**), and merged leptin-DAPI (**l**, **o**, and **r**). These are the representative images of five swine each in the VDDef and VDSuff groups and three swine in the VDSupp group. (TIF 91287 kb) Additional file 4: Figure S10. Immunofluorescent staining for TREM-2 and macrophage (CD14) and colocalization of TREM-2 with CD14^+^ cells in infrapatellar fat and muscle. Immunofluorescent staining for TREM-2 (**a**, **e**, **i**, **m**, **q**, and **u**), CD14 (**b**, **f**, **j**, **n**, **r**, and **v**), and DAPI (**c**, **g**, **k**, **o**, **s**, and **w**) was performed, and stains were merged to examine colocalization of TREM-2 and CD14 (**d**, **h**, **l**, **p**, **t**, and **x**). *Arrows* show the colocalization of TREM-2 with CD14. These are the representative images of five swine each in the VDDef and VDSuff groups and three swine in the VDSupp group. (TIF 159298 kb) Additional file 5: Figure S11. Immunofluorescent staining for TREM-2 and macrophage (CD14) as well as colocalization of TREM-2 with CD14^+^ cells in tendon and ligament. Immunofluorescent staining for TREM-2 (**a**, **e**, **i**, **m**, **q**, and **u**), CD14 (**b**, **f**, **j**, **n**, **r**, and **v**), and DAPI (**c**, **g**, **k**, **o**, **s**, and **w**) was performed, and stains were merged to examine colocalization of TREM-2 and CD14 (**d**, **h**, **l**, **p**, **t**, and **x**). *Arrows* show the colocalization of TREM-2 with CD14. These are the representative images of five swine each in the VDDef and VDSuff groups and three swine in the VDSupp group. (TIF 157582 kb) Additional file 6: Figure S12. Immunofluorescent staining for TREM-2 and macrophage (CD14) and for colocalization of TREM-2 with CD14^+^ cells in menisci and suprapatellar fat. Immunofluorescent staining for TREM-2 (**a**, **e**, **i**, **m**, **q**, and **u**), CD14 (b, f, j, n, r, v), and DAPI (**c**, **g**, **k**, **o**, **s**, and **w**) was performed, and stains were merged to examine colocalization of TREM-2 and CD14 (**d**, **h**, **l**, **p**, **t**, and **x**). *Arrows* show the colocalization of TREM-2 with CD14. These are the representative images of five swine each in the VDDef and VDSuff groups and three swine in the VDSupp group. (TIF 157634 kb) Additional file 7: Figure S13. Immunofluorescent staining for TREM-1 and macrophage (CD14) and for colocalization of TREM-1 with CD14^+^ cells in articular cartilage, tendon, ligament, and suprapatellar fat. Immunofluorescent staining for TREM-1 (**a**, **e**, **i**, and **m**), CD14 (**b**, **f**, **j**, and **n**), and DAPI (**c**, **g**, **k**, and **o**) was performed, and stains were merged to examine colocalization of TREM-1 and CD14 (**d**, **h**, **l**, and **p**). *Arrows* show the colocalization of TREM-1 with CD14. These are the representative images of five swine each in the VDDef and VDSuff groups and three swine in the VDSupp group. (TIF 107810 kb) Additional file 8: Figure S14. Immunofluorescent staining for collagen types I, II, and III in articular cartilage. Immunofluorescent staining for collagen I (**a**), collagen II (**d**), collagen III (**g**), and DAPI (**b**, **e**, and **h**). Merged images are also shown (**c**, **f**, and **i**). These are the representative images of five swine each in the VDDef and VDSuff groups and three swine in the VDSupp group. (TIF 79516 kb) Additional file 9: Figure S15. Immunofluorescent staining for MMPs 1, 2, 8, 9, and 13 in articular cartilage. Immunofluorescent staining for MMP-1 (**a**), MMP-2 (**d**), MMP-8 (**g**), MMP-9 (**j**), MMP-13 (**m**), and DAPI (**b**, **f**, **h**, **k**, **n**, and **q**), as well as merged images (**c**, **f**, **i**, **l**, **o**, and **r**), are shown. These are the representative images of five swine each in the VDDef and VDSuff groups and three swine in the VDSupp group. (TIF 75620 kb)

## Abbreviations

DAPI: 4′,6-Diamidino-2-phenylindole
H&E: Hematoxylin and eosin
IHC: Immunohistochemical
IPF: Infrapatellar fat
MFI: Mean fluorescence intensity
MMP: Matrix metalloproteinase
OA: Osteoarthritis
RA: Rheumatoid arthritis
SPF: Suprapatellar fat
TREM: Triggering receptor expressed on myeloid cells
VDDef: Vitamin D-deficient
VDR: Vitamin D receptor
VDSuff: Vitamin D-sufficient
VDSupp: Vitamin D-supplemented
