# Supplementary material for: Vitamin D attenuates inflammation, fatty infiltration, and cartilage loss in the knee of hyperlipidemic microswine
Source: Arthritis Res Ther. 2016 Sep 13;18(1):203. doi: 10.1186/s13075-016-1099-6 (PMC5022245; doi:10.1186/s13075-016-1099-6)
Supplement: Additional file 1: Table S2. — Baseline table for swine groups. The study included a total of 13 swine: 5 in the VDDef group, 5 in the VDSuff group, and 3 in the VDSupp group. Weight is indicated in pounds (lb). Average vitamin D3 levels were measured after 1 year of vitamin D diet intervention. Chondrocyte clustering was measured at × 40 magnification. (DOCX 13 kb) [file 13075_2016_1099_MOESM1_ESM.docx]

**Additional file 8: Table S2. Baseline table for swine groups**

| **Swine group** | **Number of swine** | **Average weight at arrival (lb)** | **Average weight at sacrifice (lb)** | **Average vitamin D3 levels at sacrifice** | **Average chondrocyte clustering** |
| --- | --- | --- | --- | --- | --- |
| **VD Def** | 5 | 28.95 ± 5.29 | 95.96 ± 4.53 | 8.1±1.13 ng/ml | ≈50% |
| **VD Suff** | 5 | 28.95 ± 5.29 | 112.24 ± 6.23 | 24.01±0.72 ng/ml | ≈50% |
| **VD Supp** | 3 | 35.05 ± 3.17 | 129.11 ± 3.97 | 52.58±5.68 ng/ ml | ≈3% to 5% |

The study included total 13 swine including; 5 swine in VDDeficient, 5 swine in VDSufficient, and 3 swine in VDSupplement group. Weight is indicated in pounds (lb). Average vitamin D3 levels were measured after one year of vitamin D diet intervention. Chondrocyte clustering was measured at 40X.
