# Supplementary material for: Vitamin D attenuates inflammation, fatty infiltration, and cartilage loss in the knee of hyperlipidemic microswine
Source: Arthritis Res Ther. 2016 Sep 13;18(1):203. doi: 10.1186/s13075-016-1099-6 (PMC5022245; doi:10.1186/s13075-016-1099-6)
Supplement: Additional file 2: Table S3. — Overview of all tissues with regard to inflammation and fatty infiltration. Inflammation in knee joint tissues (suprapatellar fat, infrapatellar fat, muscle, tendon, ligament, and menisci) was subjectively graded on the basis of the following criteria: nil = no inflammation; 1+ = occasional inflammatory cells; 2+ = few inflammatory cells; 3+ = many inflammatory cells; and 4+ = clumps or clusters of inflammatory cells. Macrophage density was calculated for inflammation in cartilage of all knee joints. Macrophage density (CD14+ cells) in all the knee joint tissues was analyzed for objective grading of inflammation (Fig. 6). Subjective grading of fatty infiltration was based on the following criteria: no fatty infiltration; minimal fatty infiltration = fatty infiltration in up to 5 % of tissue area; mild fatty infiltration = fatty infiltration in 6–25 % of tissue area; moderate fatty infiltration = fatty infiltration in 25–50 % of tissue area; and severe fatty infiltration = fatty infiltration in >50 % of tissue area. Further, the percentage of fatty infiltration was calculated (Fig. 2). (DOCX 16 kb) [file 13075_2016_1099_MOESM2_ESM.docx]

**Additional file 9: Table S3.** **Overview of all tissues in regards to inflammation and fatty infiltration**.

| **Swine Group** | **Infra-patellar fat** | **Supra-patellar fat** | **Muscle** | | **Tendon** | | **Ligament** | | **Menisci** | **Synovial membrane** |
| --- | --- | --- | --- | --- | --- | --- | --- | --- | --- | --- |
|  | Inflammation | Inflammation | Inflammation | Fatty Infiltration | Inflammation | Fatty Infiltration | Inflammation | Fatty Infiltration | Inflammation | Inflammation |
| **VDD** |  |  |  |  |  |  |  |  |  |  |
| 1 | ++ | ++ | + | Moderate | +++ | Mild | ++++ | Minimal | + | ++ |
| 2 | +++ | + | Nil | Mild | ++ | Mild | ++ | Minimal | + | + |
| 3 | ++ | + | + | Mild | +++ | Mild | +++ | Mild | + | + |
| 4 | +++ | ++ | + | Moderate | + | Moderate | ++ | Minimal | + | ++ |
| 5 | +++ | + | Nil | Mild | ++ | Mild | ++ | Minimal | + | ++ |
| **VDS** |  |  |  |  |  |  |  |  |  |  |
| 1 | + | Nil | Nil | Minimal | Nil | Minimal | Nil | Nil | Nil | + |
| 2 | + | Nil | + | Mild | + | Minimal | Nil | Nil | Nil | Nil |
| 3 | + | Nil | Nil | Mild | Nil | Minimal | + | Nil | Nil | Nil |
| 4 | Nil | Nil | Nil | Mild | Nil | Minimal | + | Nil | Nil | Nil |
| 5 | + | + | Nil | Mild | + | Mild | + | Mild | Nil | + |
| **VDSupp** |  |  |  |  |  |  |  |  |  |  |
| 1 | Nil | Nil | Nil | Mild | ++ | Minimal | + | Nil | Nil | Nil |
| 2 | Nil | Nil | Nil | Mild | Nil | Mild | + | Nil | Nil | Nil |
| 3 | Nil | Nil | Nil | Mild | Nil | Minimal | Nil | Nil | Nil | Nil |

Inflammation in knee joint tissues (supra-patellar fat, infra-patellar fat, muscle, tendon, ligament and menisci) was subjectively graded on following criterion: nil- no inflammation; 1+ -occasional inflammatory cells, 2+ -few inflammatory cells; 3+ -many inflammatory cells and 4+ -clumps or clusters of inflammatory cells. For inflammation in cartilage of all knee joints, macrophage density was calculated. For objective grading of the inflammation, macrophage density (CD14+ cells) in all the knee joint tissues was analyzed (Figure 6). Subjective grading of fatty infiltration was based on following criterion: no fatty infiltration; minimal fatty infiltration- fatty infiltration up to 5% of tissue area; mild fatty infiltration- fatty infiltration in 6-25% of tissue area; moderate fatty infiltration- fatty infiltration in 25-50% of tissue area and severe fatty infiltration- fatty infiltration in > 50% of tissue area. Further the percent fatty infiltration was calculated and represented as percent fatty infiltration (Figure 2).
